# Postoperative pain management

**DOI:** 10.5812/kowsar.22287523.1810

**Published:** 2011-07-01

**Authors:** Farnad Imani

**Affiliations:** 1 Department of Anesthesiology and Pain Medicine, Rasoul-Akram Medical Center, Tehran University of Medical Sciences, Tehran, IR Iran

**Keywords:** Pain, Analgesics, Anesthetics

The practice of modern anesthesiology has been developed from intraoperative period into perioperative period. Postoperative pain management is one of the most important components of adequate post-surgical patients care. This article wrote with the aim of emphasis on importance and effectiveness of post-operative pain management. Reading this article is beneficial for physicians, interventional pain managers and who care about pain medicine. Unrelieved acute pain after surgery usually elicits pathophysiologic neural alterations, including not only peripheral but also central sensitization which evolves into chronic pain syndromes. The main purpose of perioperative pain control is providing an adequate comfort level and acceptable side effects for patients. Effective postoperative analgesia improves patients’ outcome as observed by early ambulation, decrease in side effects, and reduce the incidence of postoperative chronic pain (1-3)

Even though postoperative pain management and its implications have gained a significant attention in health care during last three decades, it continues to be a major challenge that still remains disregarded (4, 5). Postoperative analgesia has traditionally been provided by administration of opioid analgesics. However, excessive opioids administration is associated with a variety of side effects including ventilatory depression, drowsiness and sedation, nausea and vomiting, pruritus, ileus, urinary retention, and constipation. Prescription of multi-modal analgesic regimens contains non-opioid analgesics (e.g., local anesthetics, nonsteroidal anti-inflammatory drugs, cyclooxygenase inhibitors, acetaminophen, ketamine, clonidine, dexmedetomodine, gabapentin) as supplement of opioid analgesics can provide better postoperative pain management outcome. The opioid-sparing effects of these compounds may lead to reduced side effects of opioids (6). Nowadays variety of new drugs, analgesic techniques and devices, and preventive approaches are available for anesthesiologists, including patient-controlled analgesia (PCA), multimodal analgesia and pre-emptive analgesia.

Besides, one of the most common methods for postoperative pain relief is PCA. This device is commonly assumed to imply on-demand intermittent, intravenous administration of opioids under patient control (with or without a continuous background infusion). PCA device is based on the use of a sophisticated microprocessor-controlled infusion pump that delivers a preprogrammed dosage of opioid analgesics when the patient pushes a demand button. Grass presented a more enlightened concept of PCA, noting that using any analgesic drugs under control of patient by any routes could be categorized as PCA, like patient-controlled epidural analgesia (PCEA) and patient-controlled regional analgesia (PCRA) (7). He proposed practical guidelines for the clinical usage of PCA, highlighted the complications and their management.

To optimize the management of acute postoperative pain, basic mechanisms of postoperative pain must be explored and new treatments must continue to be developed. Tissue damages during surgery leads to two alterations in the responsiveness of the nociceptive system, peripheral sensitization and central sensitization. Pharmacological and non-pharmacological postoperative pain management should be started quickly to suppress the development of both peripheral and central sensitization, which involves both the primary afferent nociceptors and spinal dorsal horn neurons. Understanding the neuropharmacology of the spinal cord gives us the unbelievable opportunity to base clinical management on identified mechanisms of pain receptors, pathways, and mechanisms of action.

Furthermore, evidence-based practice guidelines have the potential to provide valuable information to physicians and their patients. These guidelines not only provide guidance in routine practice, but they provide the “standard of care” for the specialists. Practice guidelines for anesthesiology and pain medicine must be improved by experts in these fields using the best available data obtained from a comprehensive review of the peer-reviewed medical literature.Anesthesiology and Pain Medicine, the official journal of Iranian Society of Regional Anesthesia and Pain Medicine (ISRAPM), aims at publishing of the scientific articles submitted by all the researchers and professionals in the field of anesthesiology and pain medicine from all over the world. It would be our pleasure to take our new steps toward medical excellence.
